# Targeted first-line therapies for advanced colorectal cancer: a Bayesian meta-analysis

**DOI:** 10.18632/oncotarget.20185

**Published:** 2017-08-11

**Authors:** Yassine Ridouane, Gilberto Lopes, Geoffrey Ku, Hasan Masud, Benjamin Haaland

**Affiliations:** ^1^ H. Milton Stewart School of Industrial and Systems Engineering, Georgia Institute of Technology, Atlanta, GA, USA; ^2^ Sylvester Comprehensive Cancer Center, University of Miami Health System, Miami, FL, USA; ^3^ Memorial Sloan Kettering Cancer Center, New York, NY, USA; ^4^ Population Health Sciences and Huntsman Cancer Institute, University of Utah, Salt Lake City, UT, USA

**Keywords:** Bayesian, colorectal cancer, decision analysis, meta-analysis, targeted therapy

## Abstract

**Background:**

Colorectal cancer is common and deadly. First-line treatments for patients with metastatic disease include FOLFIRI and FOLFOX, which have been combined with anti-EGFR or anti-VEGF antibodies to achieve benefit in selected populations. However, optimal therapy remains unclear.

**Results:**

Fifteen publications on 10 trials were identified. There was a lack of decisive evidence that FOLFIRI or FOLFOX impact efficacy of either anti-EGFR or anti-VEGF, across mutational status groups. On the other hand, evidence suggests both anti-EGFR and anti-VEGF may be more effective for *KRAS* WT than MT patients. *KRAS* WT results provided evidence that anti-EGFR treatments may be more effective than anti-VEGF treatments *when combined with FOLFIRI or FOLFOX*. Further, evidence suggests that both anti-EGFR and anti-VEGF therapies, *when combined with FOLFIRI or FOLFOX*, may be harmful as compared to chemotherapy for *KRAS* MT patients.

**Materials and Methods:**

Literature was searched for randomized trials comparing anti-EGFR or anti-VEGF antibodies, paired with FOLFIRI or FOLFOX, as first-line therapy for advanced colorectal cancer. Meta-estimates were generated via Bayesian hierarchical log-linear model. The primary endpoint was overall survival.

**Conclusions:**

Further studies examining impact of all-*RAS* mutation status, left or right side location of primary tumor, and combination anti-VEGF with modern bolus fluoropyrimidine are needed.

## INTRODUCTION

Colorectal cancer is a relatively common and deadly cancer, accounting for around 10% of both incident cancers and cancer mortalities in both women and men globally [1].

Risk factors can be non-modifiable, including age, a personal or family history of adenomatous polyps, and inflammatory bowel diseases, or modifiable, including diet, physical inactivity, obesity, smoking, and alcohol [2]. In fact, some evidence suggests that as much as 70% of colon cancer is attributable to diet and lifestyle [3].

While prognosis has progressively improved over time, survival remains poor among patients presenting with advanced or metastatic disease, with a 5-year survival rate of only 10% [2]. Unfortunately, up 20% of colorectal cancer patients present with distant metastases [4].

Typical treatments for advanced or metastatic colorectal cancer include irinotecan or oxaliplatin combined with fluorouracil and folinic acid, given infusionally, for example FOLFIRI or FOLFOX, or as a bolus, for example CAPIRI or CAPOX. More recently, these chemotherapy regimens have been combined with anti-EGFR and anti-VEGF monoclonal anti-bodies cetuximab, panitumumab, and bevacizumab [5, 6].

Cetuximab and panitumumab bind to growth factor receptors on the surface of cells, and thereby block certain types of signals causing cell division. Unfortunately, evidence suggests that *RAS*, *KRAS*, and *BRAF* mutations may side-step this mechanism of blocking uncontrolled cell division [7, 8]. Moreover, the schedule of fluoropyrimidine may influence efficacy of anti-EGFR therapies, adding complexity to treatment choice [9]. Bevacizumab, on the other hand, inhibits the function of VEGF, which stimulates new blood vessel growth. The influence of *RAS*, *KRAS*, and *BRAF* mutations and optimal schedule of fluoropyrimidine remains unclear for anti-VEGF therapy. While anti-EGFR and anti-VEGF therapies have been *combined* in a number of studies, most evidence suggests that anti-EGFR in combination with anti-VEGF therapy is inferior to either anti-EGFR or anti-VEGF alone with chemotherapy [10–13].

This meta-analysis compares anti-EGFR (cetuximab, panitumumab) therapies to anti-VEGF (bevacizumab) therapies when combined with FOLFIRI or FOLFOX chemotherapy, as first-line treatment for advanced or metastatic colorectal cancer. In order to appropriately compare anti-EGFR to anti-VEGF, the impacts of potentially important effect modifiers, choice of FOLFOX vs. FOLFIRI and mutational status (all-*RAS*, *KRAS*, *BRAF*), are also assessed.

## RESULTS

### Studies

**Figure 1:**
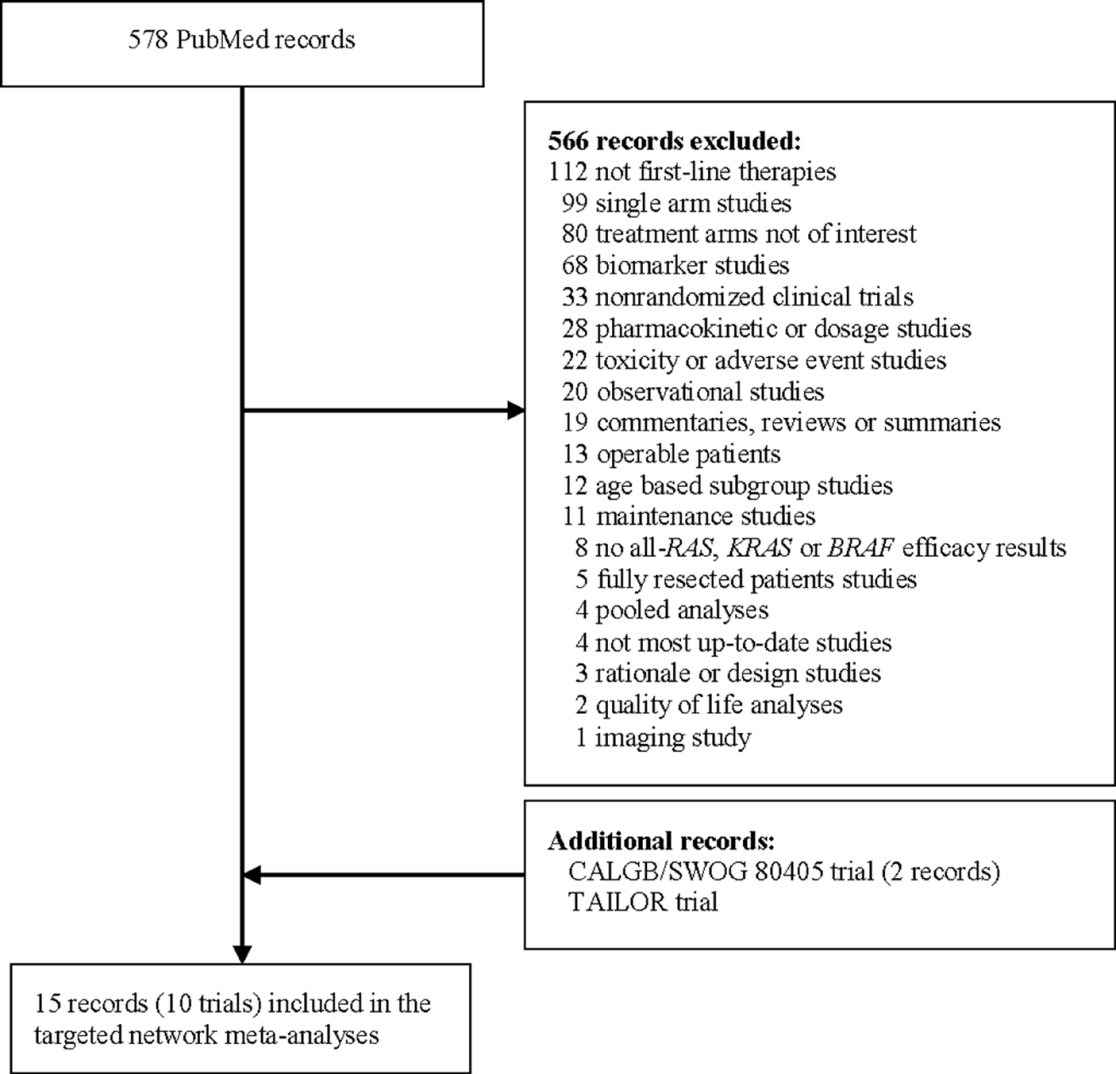
Search diagram for randomized clinical trials comparing FOLFOX or FOLFIRI chemotherapy, potentially paired with anti-EGFR (cetuximab, panitumumab) or anti-VEGF (bevacizumab) treatments, as first-line therapy for patients with inoperable advanced or metastatic colorectal cancer

PubMed [14] search identified 578 potentially relevant publications. Three additional records on two trials, CALGB/SWOG 80405 [15, 16] and TAILOR [17], were identified via supplemental searches of ASCO, GI ASCO, ESMO, and ECCO, and added to the initial list. After screening and review, 15 publications on 10 trials were identified with relevant data for at least one of the three endpoints (OS, PFS and ORR) within all-*RAS*, *KRAS*, or *BRAF* mutation status subgroups, and these formed the data for meta-analysis (Figure 1).

For the main endpoint OS, the included studies were CRYSTAL [6, 18], OPUS [19, 20], FIRE-3 [21, 22], PEAK [23], PRIME [8, 24], CECOG/CORE 1.2.001 [25], AIO KRK 0306 [26], CALGB/SWOG 80405 [15, 16] and TAILOR [17]. Each study contained two arms comparing either FOLFOX (bolus and infusional 5-FU, leucovorin and oxaliplatin) or FOLFIRI (bolus and infusional 5-FU, leucovorin and irinotecan), paired with anti-EGFR (cetuximab, panitumumab) or anti-VEGF (bevacizumab) antibodies as first-line therapy for patients with inoperable or metastatic colorectal cancer. The remaining study ITACa [27] had relevant data for PFS and ORR. Notably, the studies AVF2192g [28], MAX [29–31], AVEX [32], Saltz et al. [33] and Loupakis et al. [34] were excluded from the meta-analysis as their treatment arms either lacked oxaliplatin and irinotecan, or included both.

Table 1 summarizes studies included for meta-analysis of the main endpoint, OS, including treatment arms, effect estimates for subgroups defined by all-*RAS*, *KRAS*, or *BRAF* mutation status, and sample sizes within comparator arms. Supplementary Table 1 and 2 summarize studies included for meta-analysis of PFS and RR, respectively.

**Table 1:** Characteristics of studies included for meta-analysis of OS including patient population, treatment arms, and effect estimates (HRs along with 95% confidence intervals and sample sizes for comparator groups) for subgroups defined by all-*RAS, KRAS*, or *BRAF* mutation status

**Trial**	**Treatments (*N*)**	**All–*RAS* WT**	**Any *RAS* MT**	***KRAS* WT**	***KRAS* MT**	***BRAF***^c^ **WT**	***BRAF***^c^ **MT**
CRYSTAL [6, 18]	FOLFIRI + cetuximab (599)	0.69 (0.54–0.88) 178:189	1.05 (0.86–1.28) 246:214	0.80 (0.67–0.95) 316:350	1.03 (0.83–1.28) 214:183	0.70 (0.54–0.91) 156:159	–
	FOLFIRI^a^ (599)						
OPUS [19, 20]	FOLFOX + cetuximab (169)	0.94 (0.56–1.56) 38:49	1.29 (0.91–1.84) 92:75	0.85 (0.60–1.22) 82:97	1.29 (0.87–1.91) 77:59	0.95 (0.55–1.64) 34:45	–
	FOLFOX^a^ (168)						
FIRE-3 [21, 22]	FOLFIRI + cetuximab (297)	0.70 (0.54–0.90) 199:201	–	0.77 (0.62–0.96) 297:295	–	–	–
	FOLFIRI + bevacizumab^a^ (295)						
PEAK [23]	FOLFOX + panitumumab (142)	0.63 (0.39–1.02) 88:82	–	0.62 (0.44–0.89) 142:143	–	–	–
	FOLFOX + bevacizumab^a^ (143)						
PRIME [8, 24]	FOLFOX + panitumumab (546)	0.78 (0.62–0.99) 259:253	1.25 (1.02–1.55) 272:276	0.88 (0.73–1.06) 325:331	1.17 (0.95–1.45) 221:219	0.74 (0.57–0.96) 228:218	0.90 (0.46–1.76) 24:29
	FOLFOX^a^ (550)						
CECOG/ CORE1.2.001 [25]	FOLFOX + cetuximab (77)	–	–	0.48^d^ (0.26–0.90) 34:23		–	
	FOLFIRI + cetuximab (74)			0.74^d^ (0.39–1.40) 28:32			
AIO KRK 0306 [26]	FOLFIRI + cetuximab (50)	–	–	–	0.86 (0.55–1.35) 50:46	–	–
	FOLFIRI + bevacizumab^a^ (46)						
CALGB-SWOG 80405 [15]	Chemotherapy^b^ + cetuximab (578)	0.88^e^ (0.72–1.08) 270:256	–	0.88^f^ (0.77–1.01) 578:559	–	–	–
	Chemotherapy^b^ + bevacizumab^a^ (559)						
TAILOR [27]	FOLFOX + cetuximab (193)	0.76 (0.61–0.96) 193:200	–	–	–	–	–
	FOLFOX^a^ (200)						

^a^Reference arm; ^b^FOLFOX or FOLFIRI; ^c^*BRAF* evaluated within *RAS* WT subgroup; ^d^The reported HR is for WT compared to MT for each arm; ^e^For FOLFOX and FOLFIRI, the respective HRs are 0.86 (95% CI 0.6–1.1) 198:192 and 1.1 (95% CI 0.7–1.6) 72:64; ^f^For FOLFOX and FOLFIRI, the respective HRs are 0.83 (95% CI 0.71–0.98) 426:409 and 1.04 (95% CI 0.79–1.35) 152:150.

### Chemotherapy regimens

First, the impact of FOLFOX or FOLFIRI on the efficacy of anti-EGFR and anti-VEGF therapy is assessed within subgroups defined by all-*RAS*, *KRAS* and *BRAF* mutational status.

### All-*RAS* WT

**Figure 2:**
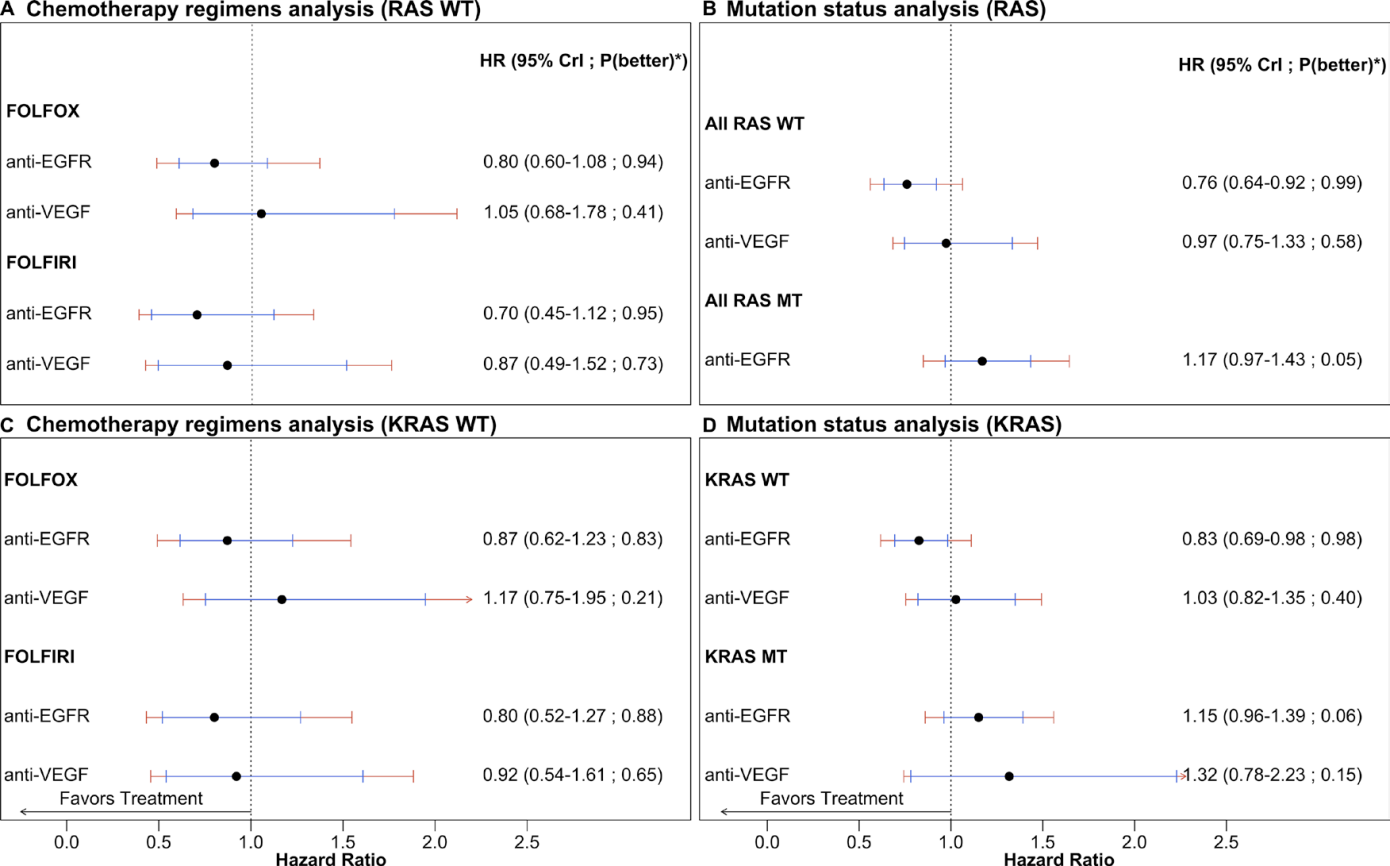
OS comparisons of anti-EGFR and anti-VEGF therapies in addition to chemotherapy regimens FOLFIRI or FOLFOX as compared to chemotherapy alone as first-line treatment for advanced or metastatic colorectal cancer (**A**) and (**C**) Chemotherapy regimen comparison for *all-RAS* and *KRAS* WT. (**B**) and (**D**) Mutation status comparison for *all-RAS* and *KRAS*. Treatments are compared to chemotherapy alone. Bayesian network estimates reported as hazard ratio (95% credible intervals in black and 95% predictive intervals in red). *Probability that the treatment arm is better than chemotherapy alone.

OS results provide *no decisive evidence* on the role of chemotherapy regimen in effectiveness of anti-EGFR or anti-VEGF treatments. Posterior median HRs for FOLFIRI as compared to FOLFOX are 0.88 (95% CrI 0.51–1.50) and 0.83 (95% CrI 0.38–1.59) respectively for anti-EGFR and anti-VEGF treatments, while respective posterior probabilities that FOLFIRI outperforms FOLFOX are 0.71 and 0.73, in the all-*RAS* WT group. See Figure 2A. Conclusions based on PFS and ORR are qualitatively similar (Supplementary Figure 1 and Supplementary Figure 2).

### *KRAS* WT

OS results for the *KRAS* WT group are broadly similar to all-*RAS* WT, with *no decisive evidence* on the role of chemotherapy in effectiveness of anti-EGFR or anti-VEGF treatments. Posterior median HRs for FOLFIRI as compared to FOLFOX within the *KRAS* WT group are 0.92 (95% CrI 0.53–1.64) and 0.79 (95% CrI 0.38–1.53) respectively for anti-EGFR and anti-VEGF treatments, while respective posterior probabilities that FOLFIRI outperforms FOLFOX are 0.65 and 0.80. See Figure 2C. Conclusions based on PFS and ORR are qualitatively similar (Supplementary Figure 1 and Supplementary Figure 2).

### *BRAF* WT within all-*RAS* WT

Similarly to all-*RAS* and *KRAS* WT, *BRAF* WT within all-*RAS* WT OS results provide *no decisive evidence* on the role of chemotherapy in effectiveness of anti-EGFR treatments. For patients who are both *BRAF* and all-*RAS* WT, posterior median HR for anti-EGFR therapy combined with FOLFIRI as compared to FOLFOX is 0.89 (95% CrI 0.42–1.85) while the posterior probability that FOLFIRI outperforms FOLFOX is 0.66. Conclusions for *BRAF* WT within *KRAS* WT or based on PFS and ORR are qualitatively similar (results not shown). For anti-VEGF treatments, data was not available.

In *RAS* and *KRAS* MT subgroups, the impact of chemotherapy on effectiveness of anti-EGFR and anti-VEGF treatments was not assessed, as evidence suggests that anti-EGFR treatments may be harmful for these patients (see below) and there was not sufficient data to assess the role of chemotherapy in effectiveness of anti-VEGF treatments.

### All-*RAS*, *KRAS*, and *BRAF* mutations

Next, the impact of all-*RAS*, *KRAS*, and *BRAF* mutations on the efficacy of anti-EGFR and anti-VEGF therapy is assessed. For this analysis, chemotherapy regimens FOLFOX and FOLFIRI are taken to be similar.

### All-*RAS*

OS results yield *substantial evidence* that anti-EGFR treatments may be more effective for all-*RAS* WT patients than patients with *RAS* mutations. For anti-EGFR treatment, the posterior median HR for WT as compared to MT is 0.65 (95% CrI 0.51–0.82), while the posterior probability that anti-EGFR treatments perform better in all-*RAS* WT than MT patients is 1.00. See Figure 2B. Conclusions based on PFS and ORR are qualitatively similar (Supplementary Figure 1 and Supplementary Figure 2). For anti-VEGF treatment, no *RAS* MT data was available and hence impact of *RAS* mutation on effectiveness could not be assessed.

### KRAS

Similarly to all-*RAS*, OS results yield *substantial evidence* that anti-EGFR treatments may be more effective for *KRAS* WT patients than patients with *KRAS* mutations. For anti-VEGF treatment, the evidence that treatment may be more effective in *KRAS* WT than MT patients is weaker. Posterior median HRs for *KRAS* WT as compared to MT are 0.72 (95% CrI 0.58–0.88) and 0.78 (95% CrI 0.45–1.39) respectively for anti-EGFR and anti-VEGF treatments, while respective posterior probabilities that treatments performed better in WT as compared to MT are 1.00 and 0.81. See Figure 2D. Conclusions based on PFS and ORR are qualitatively similar (Supplementary Figure 1 and Supplementary Figure 2).

### *BRAF* within all-*RAS* WT

Only three trials, limited to anti-EGFR compared to chemotherapy, provided evidence on the influence of *BRAF* mutation status within *RAS* WT patients. For the all-*RAS* WT group, OS results provide *no decisive evidence* on the role of *BRAF* mutation status on the effectiveness of anti-EGFR treatments as compared to chemotherapy. Within the all-*RAS* WT group, the posterior median HR for *BRAF* WT as compared to MT is 0.83 (95% CrI 0.38–1.80), while the posterior probability that anti-EGFR treatment performs better in WT as compared to MT is 0.69. Conclusions for *BRAF* WT within *KRAS* WT or based on PFS are qualitatively similar (results not shown).

### Anti-EGFR and anti-VEGF treatments

Finally, anti-EGFR and anti-VEGF therapies are compared within groups defined by *RAS* and *KRAS* mutational status. Additionally, anti-EGFR therapies (cetuximab and panitumumab) are compared within mutational groups. Once again, chemotherapy regimens FOLFOX and FOLFIRI are taken to be similar for this analysis.

### All-*RAS* WT

OS results provided *substantial evidence* that anti-EGFR treatments perform better than anti-VEGF treatments *when combined with FOLFIRI or FOLFOX*. The posterior median HR for anti-EGFR as compared to anti-VEGF is 0.78 (95% CrI 0.61–0.96) with posterior probability that anti-EGFR outperforms anti-VEGF of 0.99, in all-*RAS* WT patients. PFS and ORR results are weaker (Supplementary Figure 1 and Supplementary Figure 2).

Moreover, all-*RAS* WT OS results provide *no decisive evidence* on the impact of type of anti-EGFR treatment (cetuximab or panitumumab) on effectiveness of anti-EGFR therapy. The posterior median HR for cetuximab as compared to panitumumab is 1.05 (95% CrI 0.73–1.55), while the posterior probability that cetuximab is better than panitumumab is 0.40. Conclusions based on PFS and ORR are qualitatively similar (Supplementary Figure 1 and Supplementary Figure 2).

## *KRAS* WT

Similarly to all-*RAS* WT results, *KRAS* WT OS results also provide *substantial evidence* that anti-EGFR treatments are more effective than anti-VEGF treatments *when combined with FOLFIRI or FOLFOX*. The posterior median HR for anti-EGFR as compared to anti-VEGF is 0.80 (95% CrI 0.65–0.94), while the posterior probability that anti-EGFR outperforms anti-VEGF is 0.99, in *KRAS* WT patients. Conclusions based on ORR are qualitatively similar (Supplementary Figure 2). However, PFS results provide *no decisive evidence* of a difference (Supplementary Figure 1).

Similarly to all-*RAS* WT results, *KRAS* WT OS results provide *no decisive evidence* that type of anti-EGFR treatment (cetuximab or panitumumab) impacts effectiveness of anti-EGFR therapy. The posterior median HR for cetuximab as compared to panitumumab is 1.04 (95% CrI 0.75–1.51), while the posterior probability that cetuximab is better than panitumumab is 0.39, in the *KRAS* WT group. Conclusions based on PFS and ORR are qualitatively similar (Supplementary Figure 1 and Supplementary Figure 2).

## *KRAS* MT

OS evidence suggests that both anti-EGFR and anti-VEGF therapies may be harmful as compared to chemotherapy for patients with *KRAS* mutations. Posterior median HRs as compared to chemotherapy are 1.15 (95% CrI 0.96–1.39) and 1.32 (95% CrI 0.78–2.23) respectively for anti-EGFR and anti-VEGF treatments, while respective posterior probabilities that treatments performed better than chemotherapy are 0.06 and 0.15. See Figure 2D. Conclusions based on PFS and ORR are qualitatively similar for anti-EGFR, while they provide no decisive evidence of a difference between anti-VEGF and chemotherapy (Supplementary Figure 1 and Supplementary Figure 2).

## DISCUSSION

This meta-analysis compared anti-EGFR to anti-VEGF therapy when combined with FOLFIRI or FOLFOX chemotherapy, as first-line treatment for advanced or metastatic colorectal cancer. To appropriately compare anti-EGFR and anti-VEGF therapies, the impact of potential effect modifiers, *infusional* chemotherapy regimen (FOLFIRI, FOLFOX) and mutational status (all-*RAS*, *KRAS*, *BRAF*), were also assessed. There was a *no decisive evidence* that chemotherapy combinations FOLFIRI or FOLFOX influence effectiveness of anti-EGFR or anti-VEGF treatments across all-*RAS* and *KRAS* subgroups. On the other hand, there was *substantial evidence* that anti-EGFR therapies may be more beneficial for WT than MT patients across all-*RAS* and *KRAS* groups.

Two results in this meta-analysis were unexpected in the sense that they contradict current thinking. First, there was *substantial OS evidence* that anti-EGFR may be more beneficial than anti-VEGF for all-*RAS* and *KRAS* WT patients when combined with *infusional* fluoropyrimidine regimens FOLFIRI and FOLFOX. This result is driven by the relatively consistent findings of the FIRE-3, PEAK, and CALGB/SWOG 80405 studies. While the strength of evidence in these studies varies, all provide OS evidence that anti-EGFR may be more beneficial than anti-VEGF for *RAS* and *KRAS* WT patients when combined with *infusional* fluoropyrimidine. The role of post-progression treatments on this comparison is not clear from available data. PFS and ORR results were qualitatively similar, but with weaker evidence. Notably, there is some evidence that anti-VEGF with the *bolus* fluoropyrimidine IFL may be quite effective in the *KRAS* WT setting (anti-VEGF with IFL vs. IFL alone HR 0.6, 95% CI 0.3–1.0) [35]. In fact, an exploratory analysis comparing anti-VEGF with IFL to anti-EGFR with *infusional* fluoropyrimidine (which evidence suggests may be optimal [9]) gives strong evidence favoring anti-VEGF with IFL (data not shown).

Second, OS results suggest that in a *KRAS* mutation positive setting, adding anti-VEGF to FOLFIRI or FOLFOX may lead to inferior outcomes as compared to FOLFIRI or FOLFOX alone. The evidence that anti-VEGF combined with *infusional* fluoropyrimidine may be harmful in *KRAS* MT patients must be interpreted cautiously. This result is driven by a reasonably sizable body of evidence that *anti-EGFR* therapy may be harmful in *KRAS* MT patients, combined with relatively weak evidence from the AIO KRK-0306 study that anti-VEGF with FOLFIRI may be similar or possibly even worse [26] and fairly moderate levels of study-to-study heterogeneity. Once again, the role of post-progression treatments on this comparison is not clear. However, PFS and ORR results provide no decisive evidence that anti-VEGF combined with FOLFIRI or FOLFOX differs from chemotherapy alone (See Supplementary Figure 1 and Supplementary Figure 2). Notably, this evidence arises from an unplanned subset analysis of the AIO KRK-0306 study, which was itself a subset of the FIRE-3 study. On the other hand, there is some evidence that anti-VEGF with the *bolus* fluoropyrimidine IFL may be effective in the *KRAS* MT setting (anti-VEGF with IFL vs. IFL alone HR 0.7, 95% CI 0.4–1.3) [35]. Unfortunately, evidence from randomized trials on efficacy of anti-VEGF with more typically used bolus fluoropyrimidine regimens, such as CAPOX or CAPIRI, is not available.

The comparisons for which there was *no decisive evidence* must also be interpreted cautiously. In particular, this study found a lack of decisive evidence that chemotherapy regimen (FOLFIRI or FOLFOX) or *BRAF* mutational status (within all-*RAS* and *KRAS* WT groups) impacted effectiveness of anti-EGFR or anti-VEGF, or that type of anti-EGFR therapy (cetuximab or panitumumab) impacted effectiveness. This lack of evidence of a difference should not be misinterpreted as evidence of a lack of difference. In fact, the data are consistent with a range of possible differences favoring one side or the other (chemotherapy regimen, *BRAF* mutational status, or type of anti-EGFR therapy). From a decision analytic perspective, current evidence *does* suggest that anti-EGFR and anti-VEGF therapies may be more efficacious with FOLFIRI as compared to FOLFOX and for *BRAF* WT as compared to MT patients (within all-*RAS* and *KRAS* WT groups), and that anti-EGFR therapy panitumumab may be more efficacious than cetuximab (again, within all-*RAS* and *KRAS* WT groups), but this evidence is *very* uncertain, and in fact the data is also relatively consistent with the reverses being true.

This study has a number of limitations. All reported analyses are based on study-wise aggregated relative efficacy estimates, not individual patient data. While all included studies were of relatively high-quality with objectively reported endpoints, some analyses were greatly limited due to availability of data, particularly within all-*RAS* strata. Further, there was insufficient evidence to meta-analyze the impact on treatment efficacy of left or right primary tumor location [36]. While there was little indication of publication bias, the treatment combinations both present and absent in randomized trials reflect current clinical beliefs. Take, for example, the absence of randomized trials on anti-VEGF with modern bolus fluoropyrimidine regimens in the *KRAS* MT setting. While this study has focused on OS, and secondarily PFS and ORR, considerations such as quality of life and toxicity profile are an important component of clinical decision-making.

## MATERIALS AND METHODS

### Search strategy

Literature was searched for randomized clinical trials comparing anti-EGFR (cetuximab, panitumumab) or anti-VEGF (bevacizumab) antibodies, paired with FOLFIRI or FOLFOX chemotherapy, to one another or chemotherapy alone, as first-line therapy for patients with inoperable advanced or metastatic colorectal cancer. Medline was searched via PubMed [14] (15 February 2017) with the search phrase (“colorectal cancer” OR “colon cancer”) AND (“cetuximab” OR “panitumumab” OR “bevacizumab”), limited to publications on clinical trials. Abstracts from ASCO, GI ASCO, ESMO, and ECCO were searched for additional data. Publications were included if they contained the most up-to-date results for an endpoint of interest within all-*RAS*, *KRAS*, or *BRAF* mutation status subgroups. Risk of bias was assessed by examining patient selection and medical background, treatment arms, loss to follow-up, endpoint assessment, and reporting.

Two authors examined each paper for relevance and, if relevant, extracted comparative effectiveness summaries for endpoints of interest within all-*RAS*, *KRAS*, or *BRAF* mutation status subgroups. The primary endpoint was OS. Other endpoints of interest were PFS and ORR. Data extracted from each paper included population characteristics, number of events, HRs with corresponding 95% CIs for both OS and PFS, and ORs with the corresponding 95% CIs for ORR.

### Analysis

Meta-estimates were generated in the context of a Bayesian hierarchical log-linear model with mean effect of treatment (chemotherapy alone, chemotherapy combined with anti-EGFR or anti-VEGF) potentially depending on chemotherapy regimen, and all-*RAS*, *KRAS*, and *BRAF* mutation status subgroups. For survival outcomes, the modeled effect was the logarithm of the HR, while for ORR, the modeled effect was the logarithm of the OR. Each reported effect estimate was modeled as normally distributed centered at the relevant mean treatment comparison and having variance consisting of *within* and *between* study components.

The reciprocal of the within study variance component for study *i* was modeled as $1/\sigma_{i}^{2}∼$ Gamma $\left(D_{i}/2,\left(D_{i}/2\right)*SE_{i}^{2}\right)$, where *SE*_*i*_ was the reported (or recalculated from CIs or p-values) standard error and $D_{i}$ was the number of events for survival outcomes and the sample size for ORR. This prior for each *within* study variance is approximately centered at the study’s reported standard error and becomes more concentrated on the reported standard error as the number of events or sample size, as relevant, increases. Non-informative priors were taken for mean treatment effects as $\beta_{i}∼\left(0,{\left(\log\left(10\right)/2\right)}^{2}\right)$ for each treatment arm *i*, ensuring that approximately 95% of the prior probability was on HRs or ORs between 1/10 and 10. For *between* study standard deviations, the *weakly*-informative priors $\tau_{1}$∼Uniform (0,0.5) and $\tau_{2}$∼Uniform (0,0.8) were taken respectively for HR and OR, ensuring less than a 5% chance that an individual study’s HR or OR differed from the associated mean more than 3-fold or 5-fold respectively. In studies that did not report the number of OS or PFS events for a particular subgroup, the group’s percentage of events was estimated either as (a) the same percentage as the intention-to-treat group or (b) a fixed percentage, 60% of patients for OS and 70% of patients for PFS.

Relative treatment efficacies were summarized as posterior median HRs or ORs, along with 95% CrIs and PrIs, and posterior probability of one treatment better than the other. PrIs can be interpreted as an interval within which an HR or OR for a new study might be expected to fall. PrIs provide an intuitive assessment of uncertainty, due to both study-to-study heterogeneity and sampling error. Posterior probability one treatment better than another represents the *chance* conditional on the data that the HR for one treatment compared to another is less than one. Using the posterior probability that the treatment is best to select therapy makes sense from a decision-analytic perspective (choosing the best among equally viable alternatives). In particular, this decision rule maximizes the expected utility (other factors such as cost and quality of life taken equal). A hypothesis testing perspective, on the other hand, is biased towards the null hypothesis in the sense that the null is always selected in the absence of strong evidence. Here, *substantial evidence* was defined as a posterior median HR ≤ 0.8 (or OR ≥ 1/0.8 = 1.25) with a posterior probability ≥ 90% of the treatment outperforming the reference, while *no decisive evidence* was defined as a posterior probability of ≤ 80% of the treatment outperforming the reference [37].

All analyses were performed in R 3.0.1 (R Core Team, 2013) [38] and Markov chain Monte Carlo was performed using the rjags package [39], which calls JAGS [40] from the R environment. All Bayesian analyses were based on 10 chains, each with a 500,000 iteration burn-in followed by 500,000 posterior samples at a thinning interval of 100.

## SUPPLEMENTARY MATERIALS FIGURES AND TABLES



## Abbreviations

(CI): Confidence interval
(CrI): credible interval
(EGFR): epidermal growth factor receptor
(HR): hazard ratio
(MT): mutant
(OR): odds ratio
(ORR): objective response rate
(OS): overall survival
(PrI): predictive interval
(PFS): progression-free survival
(VEGF): vascular endothelial growth factor
(WT): wild-type
