# Supplementary material for: Targeted first-line therapies for advanced colorectal cancer: a Bayesian meta-analysis
Source: Oncotarget. 2017 Aug 11;8(39):66458–66. doi: 10.18632/oncotarget.20185 (PMC5630427; doi:10.18632/oncotarget.20185)
Supplement: Supplementary file 1 [file oncotarget-08-66458-s001.pdf]

Targeted first-line therapies for advanced colorectal cancer: a Bayesian meta-analysis

SUPPLEMENTARY MATERIALS

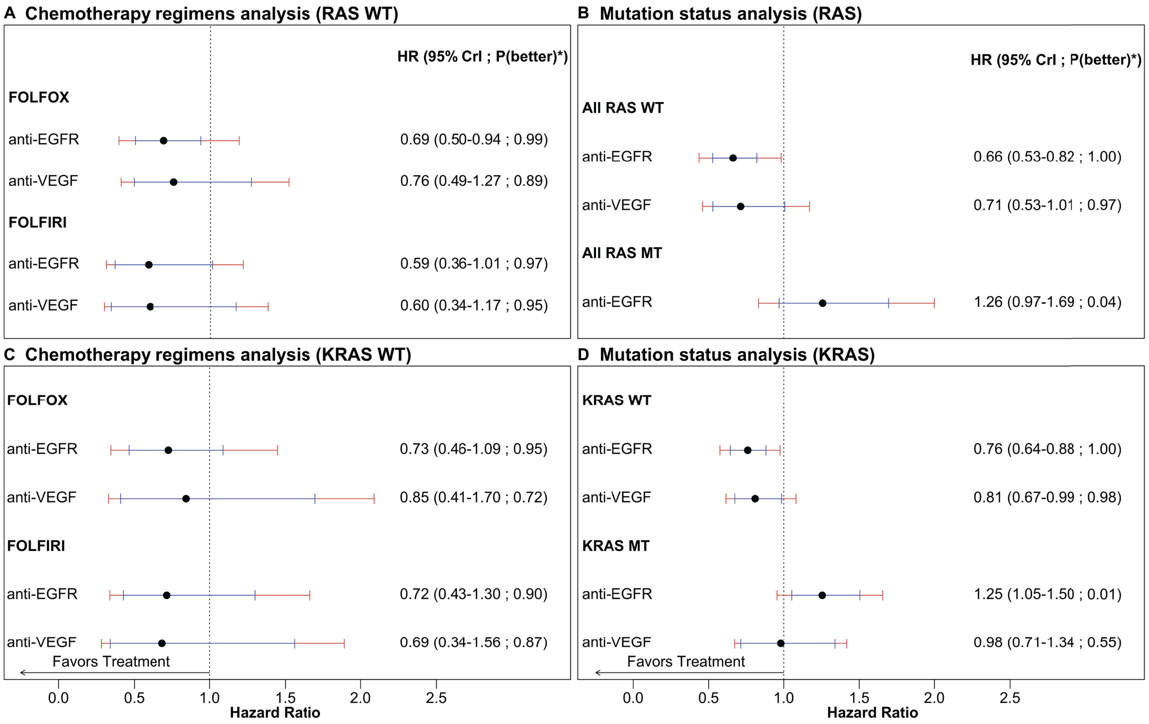

Supplementary Figure 1: PFS comparisons of anti-EGFR and anti-VEGF therapies as first-line treatment for advanced or metastatic colorectal cancer. (**A**) and (**C**) Chemotherapy regimen comparison for *all-RAS* and *KRAS* WT. (**B**) and (**D**) Mutation status comparison for *all-RAS* and *KRAS*. Treatments are compared to chemotherapy alone. Bayesian network estimates reported as hazard ratio (95% credible intervals in black and 95% predictive intervals in red). *Probability that the treatment arm is better than chemotherapy alone.

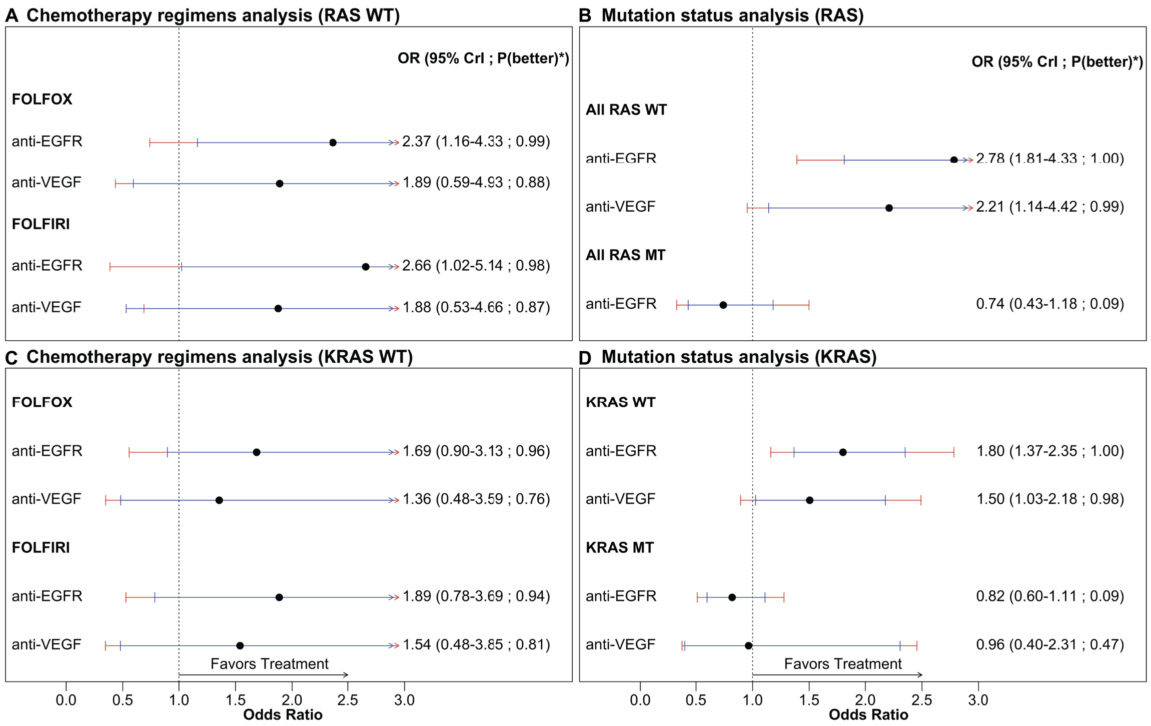

Supplementary Figure 2: ORR comparisons of anti-EGFR and anti-VEGF therapies as first-line treatment for advanced or metastatic colorectal cancer. (**A**) and (**C**) Chemotherapy regimen comparison for *all-RAS* and *KRAS* WT. (**B**) and (**D**) Mutation status comparison for *all-RAS* and *KRAS*. Treatments are compared to chemotherapy alone. Bayesian network estimates reported as odds ratio (95% credible intervals in black and 95% predictive intervals in red). *Probability that the treatment arm is better than chemotherapy alone.

Supplementary Table 1: Characteristics of studies included for meta-analysis for the secondary endpoint progression-free survival including patient population, treatment arms, and effect estimates (HRs) for subgroups defined by *KRAS*, all-*RAS*, or *BRAF* mutation status

Trial	Treatments (N)	All–*RAS* WT	Any *RAS* MT	*KRAS* WT	*KRAS* MT	*BRAF*[c] WT	*BRAF*[c] MT
CRYSTAL[1, 2]	FOLFIRI + cetuximab (599)	0.56 (0.41–0.76)	1.1 (0.85–1.42)	0.70 (0.56–0.87)	1.17 (0.89–1.54)	0.53 (0.37–0.75)	–
	FOLFIRI[a] (599)						
OPUS[3, 4]	FOLFOX + cetuximab (169)	0.53 (0.27–1.04)	1.54 (1.04–2.29)	0.57 (0.38–0.86)	1.72 (1.10–2.68)	0.51 (0.25–1.05)	–
	FOLFOX[a] (168)						
FIRE-3[5, 6]	FOLFIRI + cetuximab (297)	0.97 (0.78–1.20)	–	1.06 (0.88–1.26)	–	–	–
	FOLFIRI + bevacizumab[a] (295)						
PEAK[7]	FOLFOX + panitumumab (142)	0.65 (0.44–0.96)	–	0.87 (0.65–1.17)	–	–	–
	FOLFOX + bevacizumab[a] (143)						
PRIME[8, 9]	FOLFOX + panitumumab (546)	0.72 (0.58–0.9)	1.31 (1.07–1.6)	0.8 (0.67–0.95)	1.27 (1.04–1.55)	0.68 (0.54–0.87)	0.58 (0.29–1.15)
	FOLFOX[a] (550)						
CECOG/ CORE1.2.001[10]	FOLFOX + cetuximab (77)	–	–	0.49[d] (0.27–0.91)		–	
	FOLFIRI+ cetuximab (74)			0.66[d] (0.36–1.21)			
AIO KRK 0306[11]	FOLFIRI + cetuximab (50)	–	–	–	1.00 (0.66–1.53)	–	–
	FOLFIRI + bevacizumab[a] (46)						
CALGB-SWOG 80405[12]	Chemotherapy[b] + cetuximab (578)	1.03[e] (0.86–1.24)	–	0.95 (0.84–1.08)	–	–	–
	Chemotherapy[b] + bevacizumaba (559)						
ITACa[13]	Chemotherapy[b] + bevacizumab (176)	–	–	0.95 (0.73–1.24)	0.83 (0.58–1.18)	–	–
	Chemotherapy[a,b] (194)						
TAILOR[14]	FOLFOX + cetuximab (193)	0.69 (0.54–0.89)	–	–	–	–	–
	FOLFOX[a] (200)						

[a]Reference arm; [b]FOLFOX or FOLFIRI; [c]*BRAF* evaluated within *RAS* WT subgroup; [d]The reported HR is for WT compared to MT for each arm; [e]For FOLFOX and FOLFIRI, the respective HRs are 1.1 (95% CI 0.9–1.4) and 1.1 (95% CI 0.7–1.5).

REFERENCES

1. Van Cutsem E, Kohne CH, Lang I, Folprecht G, Nowacki MP, Cascinu S, Shchepotin I, Maurel J, Cunningham D, Tejpar S, Schlichting M, Zubel A, Celik I, et al. Cetuximab plus irinotecan, fluorouracil, and leucovorin as first-line treatment for metastatic colorectal cancer: updated analysis of overall survival according to tumor KRAS, BRAF mutation status. J Clin Oncol. 2011; 29:2011–9.
2. Van Cutsem E, Lenz HJ, Kohne CH, Heinemann V, Tejpar S, Melezinek I, Beier F, Stroh C, Rougier P, van Krieken JH, Ciardiello F. Fluorouracil, leucovorin, and irinotecan plus cetuximab treatment and RAS mutations in colorectal cancer. J Clin Oncol. 2015; 33:692–700.
3. Bokemeyer C, Bondarenko I, Hartmann JT, de Braud F, Schuch G, Zubel A, Celik I, Schlichting M, Koralewski P. Efficacy according to biomarker status of cetuximab plus FOLFOX-4 as first-line treatment for metastatic colorectal cancer: the OPUS study. Ann Oncol. 2011; 22:1535–46.
4. Bokemeyer C, Kohne CH, Ciardiello F, Lenz HJ, Heinemann V, Klinkhardt U, Beier F, Duecker K, van Krieken JH, Tejpar S. FOLFOX4 plus cetuximab treatment and RAS mutations in colorectal cancer. Eur J Cancer. 2015; 51:1243–52.
5. Heinemann V, von Weikersthal LF, Decker T, Kiani A, Vehling-Kaiser U, Al-Batran SE, Heintges T, Lerchenmuller C, Kahl C, Seipelt G, Kullmann F, Stauch M, Scheithauer W, et al. FOLFIRI plus cetuximab versus FOLFIRI plus bevacizumab as first-line treatment for patients with metastatic colorectal cancer (FIRE-3): a randomised, open-label, phase 3 trial. Lancet Oncol. 2014; 15:1065–75.
6. Stintzing S, Modest DP, Rossius L, Lerch MM, von Weikersthal LF, Decker T, Kiani A, Vehling-Kaiser U, Al-Batran SE, Heintges T, Lerchenmuller C, Kahl C, Seipelt G, et al. FOLFIRI plus cetuximab versus FOLFIRI plus bevacizumab for metastatic colorectal cancer (FIRE-3): a post-hoc analysis of tumour dynamics in the final RAS wild-type subgroup of this randomised open-label phase 3 trial. Lancet Oncol. 2016; 17:1426–34.
7. Schwartzberg LS, Rivera F, Karthaus M, Fasola G, Canon JL, Hecht JR, Yu H, Oliner KS, Go WY. PEAK: a randomized, multicenter phase II study of panitumumab plus modified fluorouracil, leucovorin, and oxaliplatin (mFOLFOX6) or bevacizumab plus mFOLFOX6 in patients with previously untreated, unresectable, wild-type KRAS exon 2 metastatic colorectal cancer. J Clin Oncol. 2014; 32:2240–7.

8. Douillard JY, Oliner KS, Siena S, Tabernero J, Burkes R, Barugel M, Humblet Y, Bodoky G, Cunningham D, Jassem J, Rivera F, Kocakova I, Ruff P, et al. Panitumumab-FOLFOX4 treatment and RAS mutations in colorectal cancer. N Engl J Med. 2013; 369: 1023–34.
9. Douillard JY, Siena S, Cassidy J, Tabernero J, Burkes R, Barugel M, Humblet Y, Bodoky G, Cunningham D, Jassem J, Rivera F, Kocakova I, Ruff P, et al. Final results from PRIME: randomized phase III study of panitumumab with FOLFOX4 for first-line treatment of metastatic colorectal cancer. Ann Oncol. 2014; 25:1346–55.
10. Ocvirk J, Brodowicz T, Wrba F, Ciuleanu TE, Kurteva G, Beslija S, Koza I, Papai Z, Messinger D, Yilmaz U, Faluhelyi Z, Yalcin S, Papamichael D, et al. Cetuximab plus FOLFOX6 or FOLFIRI in metastatic colorectal cancer: CECOG trial. World J Gastroenterol. 2010; 16:3133–43.
11. Stintzing S, Fischer von Weikersthal L, Decker T, Vehling-Kaiser U, Jager E, Heintges T, Stoll C, Giessen C, Modest DP, Neumann J, Jung A, Kirchner T, Scheithauer W, et al. FOLFIRI plus cetuximab versus FOLFIRI plus bevacizumab as first-line treatment for patients with metastatic colorectal cancer-subgroup analysis of patients with KRAS: mutated tumours in the randomised German AIO study KRK-0306. Ann Oncol. 2012; 23:1693–9.
12. Venook AP, Niedzwiecki D, Lenz H-J, Innocenti F, Fruth B, Meyerhardt JA, Schrag D, Greene C, O'Neil BH, Atkins JN. Effect of First-Line Chemotherapy Combined With Cetuximab or Bevacizumab on Overall Survival in Patients With KRAS Wild-Type Advanced or Metastatic Colorectal Cancer: A Randomized Clinical Trial. JAMA. 2017; 317:2392–401.
13. Passardi A, Nanni O, Tassinari D, Turci D, Cavanna L, Fontana A, Ruscelli S, Mucciarini C, Lorusso V, Ragazzini A, Frassineti GL, Amadori D. Effectiveness of bevacizumab added to standard chemotherapy in metastatic colorectal cancer: final results for first-line treatment from the ITACa randomized clinical trial. Ann Oncol. 2015; 26:1201–7.
14. Qin S, Xu J, Wang L, Cheng Y, Liu T, Chen J, Liu J, Li J. 169P First-line FOLFOX-4±cetuximab in patients with RAS wild-type metastatic colorectal cancer: The open-label, randomized, phase 3 TAILOR trial. Ann Oncol. 2016; 27(suppl 9).

Supplementary Table 2: Characteristics of studies included for meta-analysis for the secondary endpoint overall response rate including patient population, treatment arms, and effect estimates (ORs) for subgroups defined by *KRAS*, all-*RAS*, or *BRAF* mutation status

Trial	Treatments (N)	All–*RAS* WT	Any *RAS* MT	*KRAS* WT	*KRAS* MT	*BRAF*[c] WT	*BRAF*[c] MT
CRYSTAL[1, 2]	FOLFIRI + cetuximab (599)	3.11 (2.03–4.78)	0.85 (0.58–1.25)	2.07 (1.52–2.83)	0.82 (0.54–1.24)	3.29 (2.06–5.26)	–
	FOLFIRI[a] (599)						
OPUS[3, 4]	FOLFOX + cetuximab (169)	3.33 (1.36–8.17)	0.58 (0.31–1.08)	2.55 (1.38–4.72)	0.46 (0.23–0.92)	3.44 (1.34–8.82)	–
	FOLFOX[a] (168)						
FIRE-3[5, 6]	FOLFIRI + cetuximab (297)	1.33 (0.88–1.99)	–	1.18 (0.85–1.64)	–	–	–
	FOLFIRI + bevacizumab[a] (295)						
PEAK[7]	FOLFOX + panitumumab (142)	1.14 (0.61–2.11)	–	1.2 (0.75–1.92)	–	–	–
	FOLFOX + bevacizumab[a] (143)						
PRIME[8, 9]	FOLFOX + panitumumab (546)	–	–	1.47 (1.07–2.04)	0.98 (0.65–1.47)	–	–
	FOLFOXa (550)						
CECOG/ CORE1.2.001[10]	FOLFOX + cetuximab (77)	–	–	2.90[d] (0.95–8.84)		–	
	FOLFIRI + cetuximab (74)			1.46[d] (0.53–4.07)			
AIO KRK 0306[11]	FOLFIRI + cetuximab (50)	–	–	–	0.85 (0.37–1.98)	–	–
	FOLFIRI + bevacizumab[a] (46)						
CALGB-SWOG 80405[12]	Chemotherapy[b] + cetuximab (578)	–	–	1.20 (0.95–1.52)	–	–	–
	Chemotherapy[b] + bevacizumab[a] (559)						
TAILOR[13]	FOLFOX + cetuximab (193)	2.41 (1.61–3.61)	–	–	–	–	–
	FOLFOX[a] (200)						

[a]Reference arm; [b]FOLFOX or FOLFIRI; [c]*BRAF* evaluated within *RAS* WT subgroup; [d]The reported OR is for WT compared to MT for each arm; [e]The reported OR is for MT compared to WT.

REFERENCES

1. Van Cutsem E, Kohne CH, Lang I, Folprecht G, Nowacki MP, Cascinu S, Shchepotin I, Maurel J, Cunningham D, Tejpar S, Schlichting M, Zubel A, Celik I, et al. Cetuximab plus irinotecan, fluorouracil, and leucovorin as first-line treatment for metastatic colorectal cancer: updated analysis of overall survival according to tumor KRAS, BRAF mutation status. J Clin Oncol. 2011; 29:2011–9.
2. Van Cutsem E, Lenz HJ, Kohne CH, Heinemann V, Tejpar S, Melezinek I, Beier F, Stroh C, Rougier P, van Krieken JH, Ciardiello F. Fluorouracil, leucovorin, and irinotecan plus cetuximab treatment and RAS mutations in colorectal cancer. J Clin Oncol. 2015; 33:692–700.

3. Bokemeyer C, Bondarenko I, Hartmann JT, de Braud F, Schuch G, Zubel A, Celik I, Schlichting M, Koralewski P. Efficacy according to biomarker status of cetuximab plus FOLFOX-4 as first-line treatment for metastatic colorectal cancer: the OPUS study. Ann Oncol. 2011; 22:1535–46.
4. Bokemeyer C, Kohne CH, Ciardiello F, Lenz HJ, Heinemann V, Klinkhardt U, Beier F, Duecker K, van Krieken JH, Tejpar S. FOLFOX4 plus cetuximab treatment and RAS mutations in colorectal cancer. Eur J Cancer. 2015; 51:1243–52.
5. Heinemann V, von Weikersthal LF, Decker T, Kiani A, Vehling-Kaiser U, Al-Batran SE, Heintges T, Lerchenmuller C, Kahl C, Seipelt G, Kullmann F, Stauch M, Scheithauer W, et al. FOLFIRI plus cetuximab versus FOLFIRI plus bevacizumab as first-line treatment for patients with metastatic colorectal cancer (FIRE-3): a randomised, open-label, phase 3 trial. Lancet Oncol. 2014; 15:1065–75.
6. Stintzing S, Modest DP, Rossius L, Lerch MM, von Weikersthal LF, Decker T, Kiani A, Vehling-Kaiser U, Al-Batran SE, Heintges T, Lerchenmuller C, Kahl C, Seipelt G, et al. FOLFIRI plus cetuximab versus FOLFIRI plus bevacizumab for metastatic colorectal cancer (FIRE-3): a post-hoc analysis of tumour dynamics in the final RAS wild-type subgroup of this randomised open-label phase 3 trial. Lancet Oncol. 2016; 17:1426–34.
7. Schwartzberg LS, Rivera F, Karthaus M, Fasola G, Canon JL, Hecht JR, Yu H, Oliner KS, Go WY. PEAK: a randomized, multicenter phase II study of panitumumab plus modified fluorouracil, leucovorin, and oxaliplatin (mFOLFOX6) or bevacizumab plus mFOLFOX6 in patients with previously untreated, unresectable, wild-type KRAS exon 2 metastatic colorectal cancer. J Clin Oncol. 2014; 32:2240–7.
8. Douillard JY, Oliner KS, Siena S, Tabernero J, Burkes R, Barugel M, Humblet Y, Bodoky G, Cunningham D, Jassem J, Rivera F, Kocakova I, Ruff P, et al. Panitumumab-FOLFOX4 treatment and RAS mutations in colorectal cancer. N Engl J Med. 2013; 369:1023–34.
9. Douillard JY, Siena S, Cassidy J, Tabernero J, Burkes R, Barugel M, Humblet Y, Bodoky G, Cunningham D, Jassem J, Rivera F, Kocakova I, Ruff P, et al. Final results from PRIME: randomized phase III study of panitumumab with FOLFOX4 for first-line treatment of metastatic colorectal cancer. Ann Oncol. 2014; 25:1346–55.
10. Ocvirk J, Brodowicz T, Wrba F, Ciuleanu TE, Kurteva G, Beslija S, Koza I, Papai Z, Messinger D, Yilmaz U, Faluhelyi Z, Yalcin S, Papamichael D, et al. Cetuximab plus FOLFOX6 or FOLFIRI in metastatic colorectal cancer: CECOG trial. World J Gastroenterol. 2010; 16:3133–43.
11. Stintzing S, Fischer von Weikersthal L, Decker T, Vehling-Kaiser U, Jager E, Heintges T, Stoll C, Giessen C, Modest DP, Neumann J, Jung A, Kirchner T, Scheithauer W, et al. FOLFIRI plus cetuximab versus FOLFIRI plus bevacizumab as first-line treatment for patients with metastatic colorectal cancer-subgroup analysis of patients with KRAS: mutated tumours in the randomised German AIO study KRK-0306. Ann Oncol. 2012; 23:1693–9.
12. Venook AP, Niedzwiecki D, Lenz H-J, Innocenti F, Fruth B, Meyerhardt JA, Schrag D, Greene C, O'Neil BH, Atkins JN. Effect of First-Line Chemotherapy Combined With Cetuximab or Bevacizumab on Overall Survival in Patients With KRAS Wild-Type Advanced or Metastatic Colorectal Cancer: A Randomized Clinical Trial. JAMA. 2017; 317:2392–401.
13. Qin S, Xu J, Wang L, Cheng Y, Liu T, Chen J, Liu J, Li J. 169P First-line FOLFOX-4 ± cetuximab in patients with RAS wild-type metastatic colorectal cancer: The open-label, randomized, phase 3 TAILOR trial. Ann Oncol. 2016; 27(suppl_9).
